# Regional differences in the Association of Healthy Aging with the incidence of falls: an analysis based on the China Health and Retirement Longitudinal Study from 2011 to 2020

**DOI:** 10.3389/fpubh.2024.1416214

**Published:** 2024-08-23

**Authors:** Xiang Li, Chao Wei, Kejing Hu, Jie Sun, Xiang Gao, Jianhong Yang

**Affiliations:** ^1^Department of Neurology, The First Affiliated Hospital of Ningbo University, Ningbo, China; ^2^Ningbo Key Laboratory of Neurological Diseases and Brain Function, The First Affiliated Hospital of Ningbo University, Ningbo, China; ^3^Department of Neurosurgery, The First Affiliated Hospital of Ningbo University, Ningbo, China

**Keywords:** fall, healthy aging, middle-aged adults, urban population, rural population

## Abstract

**Background:**

Falls frequently occur among the older adult population. In this study, we examined the variations in fall incidence across different regions over time, focusing on the disparities between urban and rural areas among older adult Chinese individuals, Healthy aging is comprised of five dimensions: (1) absence of chronic diseases, (2) good physical functioning, (3) normal cognitive function, (4) active social participation, and (5) absence of depression. Additionally, we explored the relationship between healthy aging and the occurrence of falls in middle-aged and older adults. Falls are defined as events that occurred within the past two years.

**Results:**

Among 9,918 participants, 33.8% lived in urban areas and 23.0% achieved healthy aging. In contrast, 66.2% resided in rural areas with 16.5% achieving healthy aging. In 2011, rural residents had a higher fall incidence rate (17% in rural vs. 13.5% in urban); by 2020, the fall rate remained higher in rural areas (19.5% in rural vs. 17.3% in urban). Unhealthy aging (HR = 1.08, 95%CI: 1.00–1.16) were risk factors for falls. Subgroup analysis revealed that in rural areas, unhealthy aging increased the risk of falls. In urban areas, the increased risk of falls associated with unhealthy aging was not significant (Rural HR = 1.11, 95%CI:1.01–1.22; Urban HR = 1.05, 95%CI: 0.93–1.18).

**Conclusion:**

Healthy aging may be more strongly associated with a lower risk of falls in rural areas, while this association might be less pronounced in urban areas due to different environmental and social factors. This highlights the need for environment-specific fall prevention strategies and targeted measures for the older adult.

## 1 Introduction

The aging demographic is shifting globally, with individuals aged 65 and above projected to increase from 10% in 2022 to 16% by 2050 (1). This trend is particularly pronounced in Asian nations. Data from China’s Seventh National Population Census in 2020 revealed that those aged 60 and above comprised 18.3% of the total population, a 5.44% increase from the Sixth Census in 2010 (2), indicating a shift from mild to moderate aging. By 2030, the older adult population in China is anticipated to constitute about 25% (3). Reports from the World Health Organization (WHO) indicate that each year, between 28–35% of individuals aged 65 and over suffer falls, with 4–15% of these incidents resulting in severe injuries (4). In China, falls are the predominant cause of accidental injuries among the older adult, representing 52.81% of all such cases (5). The association of fall-related injuries in China is approximately double that observed in the United States (6). The repercussions of falls in the older adult include not only death and disability but also a deterioration in daily functioning, which can escalate medical costs and demand for healthcare services, rehabilitation, and support, thereby placing a significant burden on families and society (7). Given these challenges, identifying risk factors for falls and developing preventative strategies have become critical priorities for clinicians and policymakers in geriatric healthcare. Nevertheless, most existing studies on the prevalence of falls and associated factors have relied on cross-sectional designs, which constrains their ability to establish causality or demonstrate consistent correlations with falls (8, 9).

The concept of healthy aging, introduced by the WHO and defined as “the process of developing and maintaining functional ability that enables well-being in older age, “is neither novel nor exclusive but encompasses elements of successful, positive, and optimized aging (10). Research has demonstrated that healthy aging can be associated with a lower need for long-term care (11) and a decrease in all-cause mortality (12). In China, where a significant segment of the population is older adult, the importance of healthy aging is increasingly recognized. Previous research has associated unhealthy aging with conditions such as depression (13, 14); however, the connection between healthy aging and fall risk remains underexplored. As individuals age, they are more susceptible to falls due to deteriorations in normal physiological responses, vision, hearing, mobility, reflex actions, fragility, and prolonged recovery times (15). Additionally, the fear of falling can be associated with further physical decline, including reduced muscle strength and balance, thus heightening fall risk. Factors such as Parkinson’s disease, stroke, cognitive impairments, the use of sedatives and antipsychotics, joint diseases, and poor vision have been identified as contributors to impaired balance and gait (16), which in turn increase fall risk. Based on these observations, we hypothesized that healthy aging could mitigate the risk of falls. Furthermore, the residential environment (urban versus rural) influences various aspects of life, resulting in differing fall rates among these populations (17). Numerous studies have investigated these urban–rural disparities in falls (18–20), though the majority utilize cross-sectional designs that do not capture temporal changes. In this study, to generate robust findings acknowledging that falls can recur and are not isolated incidents, the Anderson-Gill model (21) was employed. This statistical approach is particularly effective for analyzing multiple instances of an event over time. This research is significant in that it extends beyond previous studies by incorporating longitudinal data to examine recurrent fall incidence rates among the older adult, thereby enhancing understanding of the factors influencing falls.

The objectives of our study are to: (1) examine the temporal variations in fall rates between urban and rural areas in China; (2) investigate the changing regional disparities in the influence of healthy aging on adult falls within China; (3) analyze whether the risk factors for falls in urban and rural settings vary over time.

## 2 Methods

### 2.1 Study design and population

**Figure 1:**
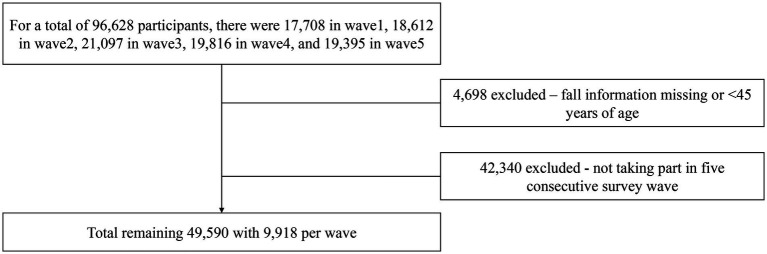
Participants selection process. Flowchart of the study participants from 2011 to 2020.

The China Health and Retirement Longitudinal Study (CHARLS) aims to collect high-quality microdata representative of Chinese households and individuals aged 45 and above (22). The national baseline survey of CHARLS was launched in 2011 and, to ensure the representativeness of the sample, covered 150 counties and 450 villages across China. A stratified random sampling method was employed to select participants from administrative regions nationwide. The sampling process involved four stages: (a) random sampling of 150 counties from all counties, excluding Tibet; (b) selection of 450 villages within these counties; (c) random sampling of household samples within these villages; and (d) random sampling of individual samples within these households. These samples are followed up every two to three years, and the data are made available to the academic community one year after the survey concludes. Initiated in 2011, CHARLS has conducted follow-up surveys in 2013, 2015, 2018, and 2020, yielding five waves of data. For a more extensive analysis, this current study incorporated data from all available waves, from wave 1 (2011) to wave 5 (2020). The inclusion criteria for our study sample required participants to be aged 45 or older and to have participated in all five waves of the survey. Additionally, subjects lacking information on falls were excluded. A total of 96,628 individuals participated from wave 1 to wave 5. After excluding 4,698 individuals under 45 years of age and 42,340 individuals who did not participate in all five waves, the final sample consisted of 9,918 individuals for each wave. The detailed inclusion and exclusion process is illustrated in Figure 1.

All CHARLS waves were ethically approved by Peking University’s Institutional Review Board (IRB number: IRB00001052-11015). Participants signed a written informed consent form.

### 2.2 Variables and covariates

#### 2.2.1 Outcomes variables

The outcome variable was defined as falls and non-falls, collected from questions in waves 1–5: “Have you fallen in the past two years?”

#### 2.2.2 Independent variables

##### 2.2.2.1 Healthy aging

The primary variable in this study is the healthy aging indicator, which is a binary variable constructed based on five dimensions. The first dimension is the absence of chronic diseases, where healthy older adults are defined as those without such conditions. The second dimension is the absence of disabilities, with healthy older adults defined as individuals who do not require assistance with activities of daily living, such as bathing, eating, dressing, moving across a room, getting in and out of bed, and using the bathroom. The third dimension is normal cognitive function, where healthy older adults in China are defined as those with “normal” cognitive abilities. The fourth dimension of healthy aging is social participation, where active engagement in social activities such as volunteering or employment is considered a positive indicator. The final component of healthy aging is the absence of depressive symptoms, with healthy older adults expected to be free of negative emotions, including feelings of fear, loneliness, or worthlessness in daily life.

1. Absence of Chronic Diseases: Healthy aging involves having none of the five common diseases in China: cancer, heart disease, lung diseases, liver and stomach diseases, and diabetes (disease count = 0). The determination of diseases is primarily conducted through interviews, during which respondents are asked about their health status and any existing conditions.
2. Good Physical Function: This is assessed through activities of daily living (ADL) and instrumental activities of daily living (IADL). ADL encompasses tasks such as dressing, incontinence management, indoor transferring, eating, toileting, and showering, while IADL covers grooming, using a phone, shopping, preparing food, housekeeping, utilizing transportation, managing medications, handling finances, and doing laundry. Participants are considered to exhibit healthy aging if they have no disabilities in ADL (ADL = 0) and no more than one disability in IADL (IADL = 0 or 1) (23).
3. Normal Cognitive Function: In the CHARLS questionnaire, cognitive function is quantified with a total score ranging from 0 to 21, consisting of the visual–spatial skills exam, the Telephone Interview for Cognitive Status (TICS-10), and episodic memory. Better cognitive performance is indicated by a higher total score (24). In this study, we use the concept of age-associated cognitive decline (AACD) to define cognitive impairment, which requires a score at least one standard deviation (SD) below the age-appropriate norm. AACD is recommended by the International Psychogeriatric Association in collaboration with the World Health Organization (WHO) as a way to identify evidence of cognitive decline across broader cognitive domains, covering all criteria for estimating cognitive impairment. Respondents are divided into age groups at five-year intervals, and individuals with cognitive scores 1 SD below the mean of their group are defined as having cognitive impairment (25).
4. Active Social Participation: Engaging in social activities such as volunteering, political activism, athletics, religion, sports, alumni relations, and non-governmental organization involvement is essential to a healthy aging process. Participating in one or more of the above described activities (social activities >1) is referred to as active social involvement (26).
5. No depression: evaluated using the Depression Scale (CESD-10) of the Center for Epidemiologic Studies (25). Participants in this study were deemed to have depressed symptoms if their depression symptom score (0–30) was less than 10 (27).

##### 2.2.2.2 Control variables

The independent factors in this study were the individuals’ physical condition, health habits, socioeconomic position, and demographics. Gender and age (45–54 years, 55–64 years, ≥165 years) are examples of demographic variables. Socioeconomic variables encompass a person’s marital status (married, single, divorced, or widowed), level of education (illiterate, primary school, junior high, high school or higher), type of health insurance, and pension status, household income (low, middle-low, middle-high, or high). Health behavior factors include smoking (yes/no), drinking (yes/no), physical activity (none/light/moderate/intense), disability (yes/no), life satisfaction (yes/no), Life satisfaction is assessed based on responses to the survey question “How satisfied are you with your life?” Responses are scored on a scale from 1 to 5, where 1 indicates “very satisfied” and 5 indicates “very dissatisfied.” These scores are then converted into a binary variable: scores of 1 to 3 are categorized as “satisfied” (yes), and scores of 4 and 5 are categorized as “dissatisfied” (no)., self-rated health (SRH) (poor/fair/good). Household income is determined based on domestic situations, where annual household income within 10,000 RMB is considered low income, 10,000–30,000 RMB as middle-low income, 30,000–80,000 RMB as middle-high income, and above 80,000 RMB as high-income households.

### 2.3 Statistical analysis

Initially, the research population’s general characteristics, falls, healthy aging, and control factors were reported using descriptive statistics, which are the frequencies and percentages for categorical variables. The overall features, control factors, and baseline components of healthy aging in urban and rural regions were compared using the chi-square test. Second, from 2011 to 2020, the incidence rates of falls in urban and rural locations were compared. Third, the Andersen-Gill model was used to examine how the incidence of falls is affected over time by healthy aging. A popular model for recurrent event survival time data, the Andersen-Gill model is essentially a straightforward expansion of the Cox proportional hazards regression model (28). It is predicated on the idea that, regardless of whether prior events have happened, the instantaneous risk of encountering an event at a time since study induction stays the same (29, 30). Falls (1 = fall) is the dependent variable in this study. Region, control factors, and healthy aging are examples of independent variables. During the course of the five follow-up evaluations in the study period, falls may repeat more than once. Based on the independent variables and elements of healthy aging, Model 1 estimated the incidence of falls from 2011 to 2020. Subgroup analysis was done in Model 2 to find geographical variations in fall-related parameters. For every model, *p*-values, 95% confidence intervals (CI), and hazardous ratios (HR) were computed. After removing all missing observations, we first carried out a thorough data analysis for the missing data. Five interpolated datasets were also produced, and multiple imputation was utilized to fill in the missing values. In this study, we employed a Mixed-Effects Generalized Linear Model (MEGLM) to analyze various risk factors influencing fall rates. Fixed effects variables include life satisfaction and unhealthy aging, while region is included as a random effects variable to account for the potential association between residential area (rural or urban) and fall rates. The MEGLM approach integrates both fixed and random effects, allowing us to address the hierarchical structure of the data and control for variability between groups. Stata (Stata Corp., College Station, TX, USA) and R (RStudio, Inc., version 4.3.1) were used for all statistical analyses, with *p*-values <0.05 denoting statistical significance.

## 3 Results

### 3.1 General characteristics and healthy aging in urban and rural areas

The baseline features of those involved are presented in Table 1, which includes 9,918 individuals (4,530 males and 5,388 women). Of these, 6,564 people, or 66.2%, were rural residents. In terms of social and individual characteristics, women made up 54.3% of the participants. 46.5% of respondents were illiterate or had only completed elementary school, with a greater prevalence of this tendency in rural areas (52.7% V.S. 34.3%, *p* < 0.001). 28.6% of participants perceived their household income as middle-high or above (annual household income ≥30,000 RMB). Regarding health behaviors, about 62.2–67.3% of participants were non-drinkers or non-smokers; 31.7% engaged in no or light physical activity. Regarding health status, 96.1% of individuals reported no physical disability.

**Table 1:** Baseline characteristics of the participants (*N* = 9,918).

Variable	Category	Total	Urban	Rural	*p*-value
Number of participants		9,918	3,354 (33.8%)	6,564 (66.2%)	
Age (years)	45–54	3,591 (36.2%)	1,285 (38.3%)	2,306 (35.1%)	0.013
	55–64	4,119 (41.5%)	1,340 (40.0%)	2,779 (42.3%)	
	≥65	2,208 (22.3%)	729 (21.7%)	1,479 (22.5%)	
Gender	Male	4,530 (45.7%)	1,509 (45.0%)	3,021 (46.0%)	0.328
	Female	5,388 (54.3%)	1845 (55.0%)	3,543 (54.0%)	
Education	Illiteracy	4,613 (46.5%)	1,151 (34.3%)	3,462 (52.7%)	< 0.001
	Primary school	2,147 (21.6%)	702 (20.9%)	1,445 (22.0%)	
	Middle school	2075 (20.9%)	871 (26.0%)	1,204 (18.3%)	
	High school and above	1,083 (10.9%)	630 (18.8%)	453 (6.9%)	
Marital status	Married	8,915 (89.9%)	3,028 (90.3%)	5,887 (89.7%)	0.349
	Unmarried/divorced/widowed	1,003 (10.1%)	326 (9.7%)	677 (10.3%)	
Physical activity intensity	No	732 (7.4%)	271 (8.1%)	461 (7.0%)	< 0.001
	Light	2,411 (24.3%)	933 (27.8%)	1,478 (22.5%)	
	Moderate	3,230 (32.6%)	1,123 (33.5%)	2,107 (32.1%)	
	Intense	3,545 (35.7%)	1,027 (30.6%)	2,518 (38.4%)	
Smoke	Yes	3,752 (37.8%)	1,196 (35.7%)	2,556 (38.9%)	0.001
	No	6,166 (62.2%)	2,158 (64.3%)	4,008 (61.1%)	
Drink	Yes	3,244 (32.7%)	1,097 (32.7%)	2,147 (32.7%)	0.999
	No	6,674 (67.3%)	2,257 (67.3%)	4,417 (67.3%)	
Household income	Low	4,802 (48.4%)	1,349 (40.2%)	3,453 (52.6%)	< 0.001
	Middle-low	2,286 (23.0%)	790 (23.6%)	1,496 (22.8%)	
	Middle-high	2,169 (21.9%)	921 (27.5%)	1,248 (19.0%)	
	High	661 (6.7%)	294 (8.8%)	367 (5.6%)	
Disability	Yes	391 (3.9%)	117 (3.5%)	274 (4.2%)	0.088
	No	9,527 (96.1%)	3,237 (96.5%)	6,290 (95.8%)	
Medical insurance	No	584 (5.9%)	276 (8.2%)	308 (4.7%)	< 0.001
	Urban employee medical insurance	792 (8.0%)	678 (20.2%)	114 (1.7%)	
	Urban and rural resident medical insurance	136 (1.4%)	75 (2.2%)	61 (0.9%)	
	Urban resident medical insurance	325 (3.3%)	279 (8.3%)	46 (0.7%)	
	New rural cooperative medical insurance	7,925 (79.9%)	1939 (57.8%)	5,986 (91.2%)	
	Government medical insurance	129 (1.3%)	88 (2.6%)	41 (0.6%)	
	Other	27 (0.3%)	19 (0.6%)	8 (0.1%)	
Pension	Yes	3,553 (35.8%)	1,649 (49.2%)	1904 (29.0%)	< 0.001
	No	6,365 (64.2%)	1705 (50.8%)	4,660 (71.0%)	
Life satisfaction	Yes	7,697 (77.6%)	2,619 (78.1%)	5,078 (77.4%)	0.411
	No	2,221 (22.4%)	735 (21.9%)	1,486 (22.6%)	
Self-reported health	Poor	2,648 (26.7%)	680 (20.3%)	1968 (30.0%)	< 0.001
	Normal	4,944 (49.8%)	1795 (53.5%)	3,149 (48.0%)	
	Good	2,326 (23.5%)	879 (26.2%)	1,447 (22.0%)	
Fall	Yes	1,570 (15.8%)	452 (13.5%)	1,118 (17.0%)	< 0.001
	No	8,348 (84.2%)	2,902 (86.5%)	5,446 (83.0%)	
Healthy aging	Yes	1852 (18.7%)	771 (23.0%)	1,081 (16.5%)	< 0.001
	No	8,066 (81.3%)	2,583 (77.0%)	5,483 (83.5%)	

There were notable variations between the urban and rural participants’ levels of education, physical activity intensity, smoking habit, household income, health insurance, pensions, and SRH. The distribution of baseline healthy aging and its elements in urban and rural populations is shown in Table 2. Overall, 18.7% of participants were considered to be aging healthily, with those in urban areas at 23.0% and those in rural areas at 16.5%, demonstrating significant differences among baseline dwelling locations (*p* < 0.001). Among the five elements of healthy aging, physical function and cognitive function were significantly higher in urban areas. In contrast, there was a considerable difference between urban and rural populations’ levels of social involvement and mental health. The Effect of health aging on fall unadjusted model can be seen in the Supplementary Material Table S1.

**Table 2:** Description of the components of healthy aging at baseline (*N* = 9,918).

Variables		Total	Urban	Rural	*p*-value
*N*		9,918	3,354 (33.82%)	6,564 (66.18%)	
Healthy aging	No	8,066 (81.3%)	2,583 (77.0%)	5,483 (83.5%)	<0.001
	Yes	1852 (18.7%)	771 (23.0%)	1,081 (16.5%)	
Components	Definition				
Absence of chronic disease	Number of chronic				
	≥1	3,983 (40.2%)	1,368 (40.8%)	2,615 (39.8%)	0.36
	0	5,935 (59.8%)	1986 (59.2%)	3,949 (60.2%)	
Good physical function	ADL				
	0	8,406 (84.7%)	2,967 (88.5%)	5,439 (82.9%)	<0.001
	≥1	1,512 (12.3%)	387 (11.5%)	1,125 (17.1%)	
	IADL				
	0–1	8,009 (80.8%)	2,894 (86.3%)	5,115 (77.9%)	<0.001
	≥2	1909 (19.2%)	460 (13.7%)	1,449 (22.1%)	
	Sum Of ADL And IADL				
	0–1	8,058 (81.2%)	2,893 (86.3%)	5,165 (78.7%)	<0.001
	≥2	1860 (18.8%)	461 (13.7%)	1,399 (21.3%)	
Normal cognitive function	Cognitive impairment				
	Yes	1786 (18.1%)	430 (12.8%)	1,356 (20.7%)	<0.001
	No	8,132 (81.9%)	2,924 (87.2%)	5,208 (79.3%)	
Good psychological adaptation	Depression				
	Yes	3,181 (32.1%)	833 (24.8%)	2,348 (35.8%)	<0.001
	No	6,737 (67.9%)	2,521 (75.2%)	4,216 (64.2%)	
Active social engagement	Participation in one or more activities				
	Yes	4,942 (35.9%)	1773 (52.9%)	3,169 (48.3%)	<0.001
	No	4,976 (50.2%)	1,581 (47.1%)	3,395 (51.7%)	

### 3.2 Incidence of falls from 2011–2020

**Figure 2:**
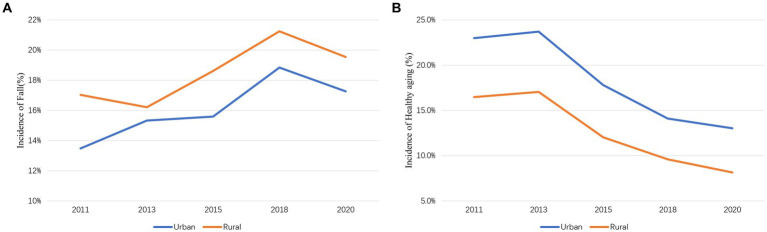
**(A)** Changes in the incidence of fall from 2011 to 2020. **(B)** Changes in the incidence of healthy aging from 2011 to 2020.

Figure 2A shows the frequency of falls from 2011 (wave 1) to 2020 (wave 5) in urban and rural areas. In 2011, rural areas experienced a higher rate of falls (17.3%) than urban areas (13.5%). Despite fluctuations, both urban and rural areas saw an increase in fall incidence rates compared to 2011, with rural areas consistently higher than urban areas. The analysis using a mixed-effects generalized linear model indicated that unhealthy aging significantly increases the risk of falls among older adults (*p* < 0.001), and life satisfaction also has a significant impact on fall risk (p < 0.001). The detailed results are provided in the attached document. Figure 2B shows the incidence of healthy aging and falls from wave 1 to wave 5. Throughout the follow-up, the rate of healthy aging declined in both urban and rural populations, with urban populations maintaining a higher rate of healthy aging (13.0%) compared to rural areas (8.2%) by the wave 5 survey.

### 3.3 Association of Healthy Aging with falls from 2011–2020

**Figure 3:**
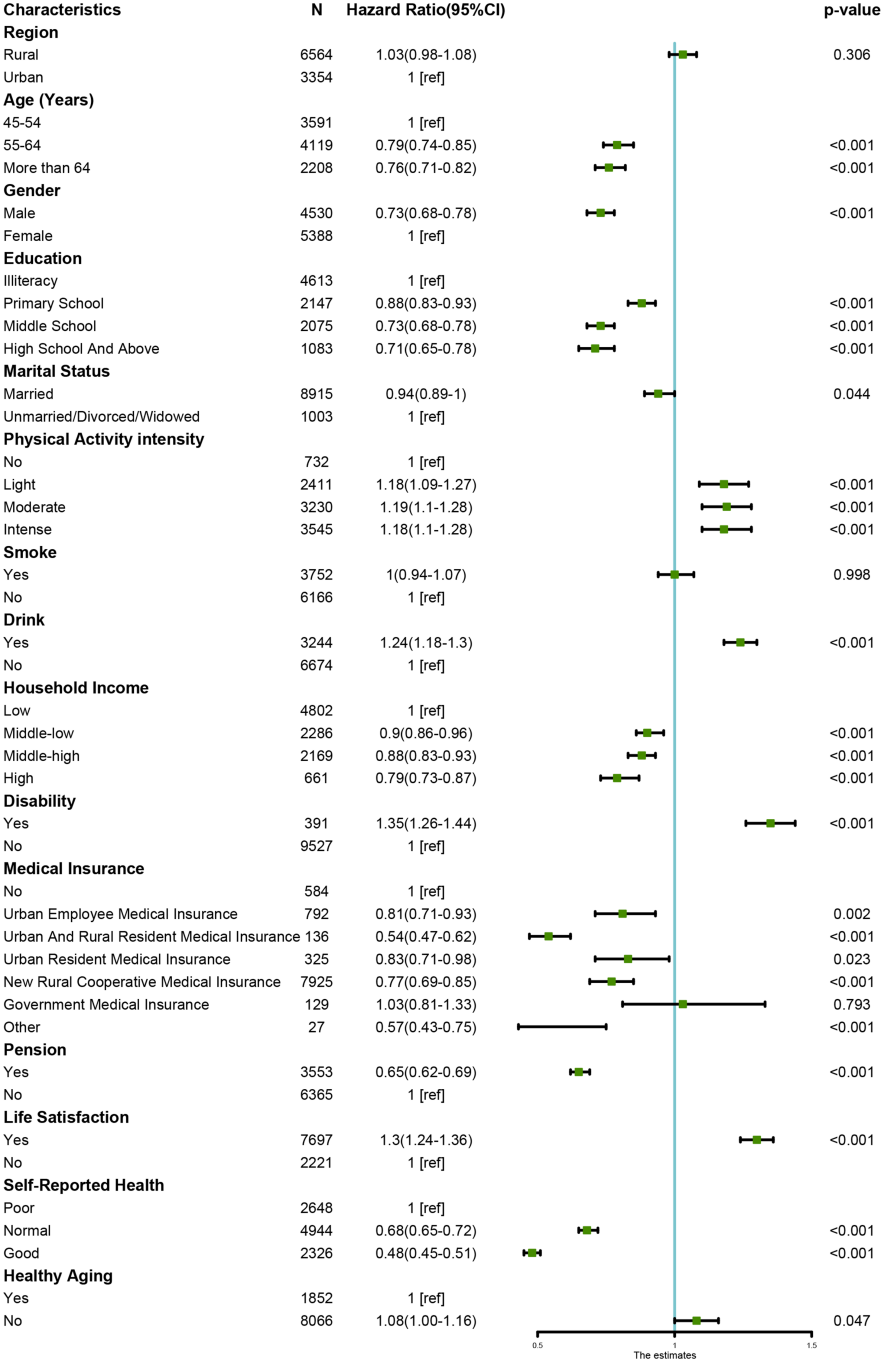
Factors related to fall.

Figure 3 displays instantaneous Cox regression results for all participants (2011–2020) to identify factors influencing falls and HRs. Among the five sub-factors of healthy aging, four are significantly related to falls: higher ADL scores, abnormal cognitive function, poorer mental health, and lower social participation are all significantly associated with falls. Additionally, being male, older age, lower education level, higher physical activity intensity, lower self-reported health status, disability, type of medical insurance, drinking behavior, absence of a pension, lower life satisfaction, lower income level, and unhealthy aging are all significant factors for falls. We conducted an additional analysis of the history of falls in Table S2 of the Supplementary Material.

### 3.4 Association of Healthy Aging on falls by residential area

The subgroup analysis findings are displayed in Table 3. Lower education levels were linked to falls during the course of the research period (2011–2020), with rural regions exhibiting a decrease in fall risk as education levels increased (HR for elementary, junior high, and high school or above, respectively: 0.88, 0.74, 0.69). Regarding household annual income, its association was notable in rural areas, with fall risk decreasing as household annual income increased (HR for primary, junior high, high school or 189 above respectively: 0.88, 0.74, 0.69). High-income families in urban populations provided protection against falls (HR = 0.82, 95% CI: 0.72–0.93); however, middle-class and lower-class persons did not show any difference in this regard. Regardless of living in an urban or rural area, people with pensions were associated with a lower risk of falling compared to those without pensions. To verify the robustness of the above model results, we conducted sensitivity analysis using blood metabolic indicators. The results are shown in Table S3 of the Supplementary Material.

**Table 3:** Regional comparison of the time-dependent Cox recurrent models between fall and related factors.

Variable	Categories	Urban		*p*-Value	Rural		*p*-Value
		HR	95% CI		HR	95% CI	
Age (years)	45–54	1.00	[ref]		1.00	[ref]	
	55–64	0.79	(0.70–0.90)	<0.001	0.79	(0.73–0.86)	<0.001
	≥65	0.73	(0.64–0.84)	<0.001	0.78	(0.71–0.85)	<0.001
Gender	Male	0.72	(0.64–0.81)	<0.001	0.73	(0.67–0.8)	<0.001
	Female	1.00	[ref]		1.00	[ref]	
Education	Illiteracy	1.00	[ref]		1.00	[ref]	
	Primary school	0.85	(0.77–0.95)	0.003	0.88	(0.83–0.94)	<0.001
	Middle school	0.70	(0.63–0.79)	<0.001	0.74	(0.68–0.8)	<0.001
	High school and above	0.72	(0.63–0.82)	<0.001	0.69	(0.61–0.78)	<0.001
Marital status	Married	0.89	(0.8–0.98)	0.022	0.97	(0.91–1.04)	0.448
	Unmarried/divorced/widowed	1.00	[ref]		1.00	[ref]	
Physical Activity intensity	No	1.00	[ref]		1.00	[ref]	
	Light	1.08	(0.93–1.24)	0.304	1.22	(1.11–1.34)	<0.001
	Moderate	0.99	(0.86–1.14)	0.864	1.30	(1.18–1.42)	<0.001
	Intense	1.25	(1.09–1.45)	0.002	1.17	(1.07–1.28)	<0.001
Smoke	Yes	1.00	(0.89–1.12)	0.999	1.00	(0.93–1.08)	0.974
	No	1.00	[ref]		1.00	[ref]	
Drink	Yes	1.22	(1.11–1.34)	<0.001	1.25	(1.17–1.32)	<0.001
	No	1.00	[ref]		1.00	[ref]	
Disability	Yes	1.30	(1.14–1.48)	<0.001	1.37	(1.26–1.49)	<0.001
	No	1.00	[ref]		1.00	[ref]	
Household income	Low	1.00	[ref]		1.00	[ref]	
	Middle-low	1.01	(0.91–1.12)	0.799	0.86	(0.81–0.92)	<0.001
	Middle-high	1.01	(0.92–1.11)	0.826	0.82	(0.77–0.88)	<0.001
	High	0.83	(0.73–0.95)	0.008	0.78	(0.7–0.88)	<0.001
Medical insurance	No	1.00	[ref]		1.00	[ref]	
	Urban employee medical insurance	0.74	(0.6–0.91)	0.004	0.78	(0.63–0.97)	0.024
	Urban and rural resident medical insurance	0.48	(0.38–0.6)	<0.001	0.57	(0.49–0.68)	<0.001
	Urban resident medical insurance	0.73	(0.59–0.91)	0.005	0.92	(0.68–1.25)	0.59
	New rural cooperative medical insurance	0.65	(0.54–0.79)	<0.001	0.82	(0.72–0.94)	0.003
	Government medical insurance	1.10	(0.8–1.52)	0.564	0.78	(0.5–1.22)	0.275
	Other	0.64	(0.42–0.95)	0.029	0.49	(0.33–0.72)	<0.001
Pension	Yes	0.68	(0.62–0.75)	<0.001	0.64	(0.6–0.68)	<0.001
	No	1.00	[ref]		1.00	[ref]	
Life satisfaction	Yes	1.28	(1.18–1.4)	<0.001	1.30	(1.23–1.38)	<0.001
	No	1.00	[ref]		1.00	[ref]	
Self-reported health	Poor	1.00	[ref]		1.00	[ref]	
	Normal	0.71	(0.65–0.78)	<0.001	0.67	(0.64–0.71)	<0.001
	Good	0.49	(0.43–0.55)	<0.001	0.48	(0.44–0.52)	<0.001
Healthy aging	Yes	1.00	[ref]		1.00	[ref]	
	No	1.05	(0.93–1.18)	0.452	1.11	(1.01–1.23)	0.032

[ref], Indicates the reference group in the analysis. The reference group serves as the baseline for comparison, and therefore, no confidence interval is reported for it.

## 4 Discussion

Unhealthy aging raised the incidence of falls in rural regions, according to a geographical analysis of falls and associated variables. In urban regions, this rise was not statistically significant (urban HR = 1.05, 95%CI: 0.93–1.18; rural HR = 1.11, 95%CI:1.01–1.22). In terms of health behaviors, in rural areas, light to intense physical activities increased the risk of falls (HR and 95%CI for light, moderate, and intense physical activity respectively: 1.22 (1.13–1.35); 1.30 (1.18–1.42); 1.17 (1.07–1.28)). In urban areas, light to moderate physical activity was not significantly associated with falls, whereas intense physical activity was associated with an increased risk of falls (HR = 1.25, 95%CI: 1.09–1.45). Higher self-assessment of health was associated with a lower risk of falls in both urban and rural locations.

In order to better understand the connection between falls and healthy aging, this study examined longitudinal data on the health of middle-aged and older persons in China from 2011 to 2020, paying particular attention to regional variations within the country. The risk of falls is 1.08 times higher in the unhealthy aging people than in the healthy aging group, according to our model analysis, which shows that fall incidence rates are higher in rural areas than in urban areas, while the differences are not statistically significant.

According to the classic definition by Rowe and Kahn (31), successful aging is characterized by minimal disease and disability, high levels of cognitive and physical function, and an active lifestyle in later years. However, as research on aging has progressed, definitions of healthy aging have varied. An analysis of 27 studies on successful aging found that the reported prevalence of successful aging ranged from 0.4 to 95.0% (32). This inconsistency primarily arises from the lack of a widely accepted definition and measurement framework for successful aging. Researchers increasingly recognize that successful aging should be multidimensional, encompassing not only biomedical factors but also social and psychological aspects (33, 34). In 2015, the World Health Organization (WHO) introduced the concept of healthy aging, defining it as “the process of developing and maintaining the functional ability that enables well-being in older age.” Healthy aging is not a new concept but rather an inclusive one related to successful, active, and he optimized aging. This paper adopts a comprehensive definition of healthy aging (HA) based on several classic sources and adapted to the context of China (23, 32, 35). The study focuses on five dimensions selected for their relevance to exploring the association of healthy aging. However, due to differences in cultural background and economic development, our current definition may not fully capture the true state of healthy aging. Addressing this issue will be a focus of future research.

In our longitudinal analysis, we found that fall rates among middle aged and older adult individuals in urban and rural region have generally trended upwards. Previous research confirms this, showing that the risk of falls increases with age due to factors such as impaired balance, decreased mobility, and declines in vision and cognitive function (36). In addition, physical and physiological characteristics make older people more vulnerable to unintentional fall-related accidents (37). Therefore, the increase in fall rates among the older adult may be related to the decline in physical function. However, we also observed a decrease in fall rates in both urban and rural populations in the 2020 survey. The CHARLS 2020 survey (wave5), conducted from 2020 to 2022, coincided with the COVID-19 pandemic, during which the Chinese government implemented strict pandemic control measures. For much of this period, people in both urban and rural areas were engaged in home-based activities, resulting in less movement and activity compared to before. This is indirectly associated with decreased activity levels among the older adult, the population’s fall rates correspondingly decrease, aligning with our findings on physical activity intensity. Our study found that as the intensity of physical activity increases, so does the likelihood of falls. In our study, 66.2% of participants came from rural areas, and in the CHARLS questionnaire, physical activity includes job demands, entertainment, and exercise. For most participants, the physical activity reported in the survey was related to work requirements rather than regular exercise. Previous studies have found that exercise is associated with a lower incidence of falls (38). However, physical activity and exercise differ in our study, leading to different outcomes. Thus, it is evident in our study that compared to individuals with no physical activity, those with higher intensity physical activity have higher fall rates, especially in rural populations, where many residents engage in farming and other productive activities. Even at an older age, they may assist in family farming activities. Therefore, The Chinese government should enhance public facility construction in rural areas and improve the welfare of the older adult to address reduced physical activity from farming and potentially lower fall rates.

Furthermore, our study found that over the past decade, the fall rates in rural populations have consistently been higher than those in urban populations. One study found that living conditions in rural areas, such as outdoor toilets, uneven rural roads (39), and lack of heating facilities (40), increase the risk of falls to varying degrees. In addition, squat toilets are more common in rural Chinese communities due to cultural norms and financial constraints than sitting toilets. Orthostatic hypotension is a common side effect of standing and squatting, which raises the risk of falling. It has also been demonstrated by earlier studies that falling can be more likely while using a seated toilet (41). In summary, better home environments among middle-aged and older individuals in China, including sitting toilets, clean living conditions, and the use of clean cooking fuels, may be associated with a lower risk of falls (42). More work is required to comprehend the influence of environmental variables (such as rural settings) on the association between behavior and falls in the background of each nation, as the chance of falls depends on the interplay between environmental factors and behavior (43).

Previous research has found that social support not only provides participants with systematic fall prevention training but also is associated with a reduction in their fear of falling (44), which in turn is related to a lower frequency of falls (45). The safety of the living environment, the completeness of facilities, and the friendliness of the community all influence the activity range and fall risk for older adults (42, 43, 46). Timely and appropriate medical care can be associated with a lower occurrence of falls. Easy access to medical resources, early recognition of the need for medical care, and proactive assessments may be related to a lower number of falls and a decrease in their severity (47). To address falls among older adults, community-level health education and fall prevention training can be implemented to enhance health awareness and fall prevention capabilities. Promoting suitable fitness activities for the older adult, such as Tai Chi and brisk walking, may improve physical fitness and be associated with a lower fall risk. Improving medical services in urban and rural areas and providing more preventive health services, such as regular physical examinations and fall risk assessments, are important. Strengthening chronic disease management among older adults can help manage conditions and may be associated with lower fall risks. Furthermore, creating safe living environments in urban and rural areas by addressing potential fall hazards, such as uneven pavements and staircases without handrails, is essential. Offering age-friendly renovation services to adapt the living environments of older adults can make their homes more suitable for daily life.

According to our research, older adult and middle-aged people who have less education are more likely to fall. Higher education also prioritizes preserving health, improves medication adherence, and lowers the chance of falls, according to another study (48). Our study also found that good lifestyle habits, such as not smoking, not drinking, high life satisfaction, and a high self-assessment of health, are associated with a lower rate of falls, reflecting the individual’s psychological health from various perspectives. Therefore, better psychological health may be associated with a lower rate of falls. Additionally, our study found that higher total household income is associated with a lower fall rate in both urban and rural populations, similar to the association with various types of medical insurance and pensions. By reducing out-of-pocket (OOP) costs, China’s social health insurance (SHI) plan efficiently enhances timely utilization of healthcare services, particularly for those with lower socioeconomic level (49, 50). The distribution of service consumption and insurance benefits between the affluent and the poor, however, continues to be a major indicator of the extreme inequality of access to healthcare in China (51). Numerous studies demonstrate that, if socioeconomically better off, patients from wealthy families or metropolitan regions tend to use more healthcare services and gain more from SHI reimbursements than do members of underprivileged communities (52, 53). Overall, although China provides universal medical insurance, there are still significant differences in healthcare access among different socioeconomic groups. Differences in household income, pensions, and types of medical insurance lead to variations in individuals’ attention to their health, causing disparities in fall rates between urban and rural areas.

Healthy aging was made a worldwide priority when the WHO and the UN unveiled a 10-year global action plan in 2020, known as the United Nations Decade of Healthy Aging (2021–2030) (54). However, there are no trustworthy instruments or survey methods to quantify the multifaceted and incredibly variable idea of healthy aging. There have been reports of investigations on the creation of the Healthy Aging Index recently (45). As a result, we evaluated the incidence of healthy aging using data from effective aging studies—a notion that is used with more frequency (35). Compared to the 32–35% of persons 45 and older who reported participating in the Korean KLoSA survey (13), our study’s wave 5 prevalence of healthy aging was 9.8%, which is lower. A different research with 2,157 participants from five European nations who were at least 70 years old found that 41.8% of them were healthy seniors (55). In comparison, 79% of 3,100 U.S. veterans aged 60 and above reported self-rated healthy aging (56). The variations in the criteria of healthy aging and the demographics seen in the previously stated research might account for these discrepancies. Therefore, a standardized healthy aging tool that considers cultural differences between countries should be used to monitor healthy aging.

Of the five signs of aging healthily, mental health (unadjusted HR = 1.41), ADL (unadjusted HR = 1.32), and IADL (unadjusted HR = 1.32) showed a higher risk ratio for falls than other factors. The state of one’s mental health is essential for aging in a healthful manner. Previous studies have revealed that depression symptoms are linked to both fear of falling and falling risk in the older adult (57). Falling is more likely to occur in older people with higher degrees of depressive symptoms, and falling is more frightening to people who are depressed. It’s still unclear exactly how depression symptoms and falls are related. Therefore, actively managing depressive symptoms is crucial for fall prevention (58). It need good ADL and IADL to keep one’s autonomy and independence. ADL limits have been linked to reduced gait speed and worse balance, which has been linked to an increased risk of falls (36, 59, 60). The results of the current study confirm that enhancing physical function should be a key component of health aging intervention programs (61–64). It is important to take these things into account while creating treatments for healthy aging.

In summary, healthy aging involves multiple aspects, including good physical health, active social participation, and good mental state. In rural areas, healthy aging as a protective factor against the risk of falls may be related to fewer environmental risks and closer community ties. Individuals in rural areas may have easier access to neighbor support, and in smaller communities, the older adult may be more active, maintaining better physical condition and social activities. In contrast, in urban areas, although individuals aging healthily also tend to have a lower risk of falls, this effect was not statistically significant. This may be due to the complexity of urban environments, faster pace of life, and greater environmental risks faced by the older adult, such as traffic and sidewalk conditions, which may weaken the protective effect of healthy aging on reducing the risk of falls. In rural areas, strengthening community support and establishing family support networks can enhance awareness and capabilities among older adults and their family members. Additionally, improving the supply of basic medical services in rural areas is crucial to ensure that older adults have access to timely healthcare. In urban areas, establishing senior activity centers can promote social engagement and physical exercise among older adults. Moreover, leveraging technology, such as health monitoring devices and smart home technologies, can improve safety and quality of life for the older adult.

## 5 Limitation

The study has significant limitations. This secondary data analysis does not include physical environmental factors that may be associated with falls, such as housing, neighborhood facilities, and social capital. Future study should examine how these factors influence regional disparities in depression prevalence rates. Furthermore, there is no unanimity on healthy aging. A thorough analysis of healthy aging found 65 models with varying subcomponents (ranging from 2 to 9). In addition, some frameworks consider depression as a component of healthy aging (35). This study adopted successful aging as the closest notion to healthy aging, which was also employed in the Korean KLoSA study (13). Third, information on falls may be affected by recollection bias. Our yearly survey approach of determining falls may be less accurate than more stringent methods like monthly fall diaries. Finally, the factors used in this study may vary with time. Future research should employ appropriate analytical approaches to examine how changes in healthy aging and other factors are associated with the occurrence of falls. This study recorded only participants’ fall information from the past two years and did not capture a complete history of falls or conduct a detailed assessment of fall severity. This simplified measurement approach may have overlooked some aspects of fall-related risks. Additionally, we recognize that social desirability bias could affect respondents’ answers, particularly concerning personal health and fall incidents. Some participants may have underestimated the frequency of falls due to reluctance to acknowledge them. Future research could mitigate this bias by employing various data collection methods, such as supplementary reports from family members or caregivers.

## 6 Conclusion

Overall, healthy aging may be more strongly associated with a lower risk of falls in rural areas, while in urban areas, this association may be less pronounced due to differing environmental and social factors. This emphasizes the need to consider the living environment of the older adult when developing fall prevention strategies and to take targeted measures, such as improving urban environmental safety, providing community support, and promoting health activities, to foster healthy aging.

## Data Availability

The datasets presented in this study can be found in online repositories. The names of the repository/repositories and accession number(s) can be found at: China Health and Retirement Longitudinal Study, https://charls.pku.edu.cn/.
