# Supplementary material for: Regional differences in the Association of Healthy Aging with the incidence of falls: an analysis based on the China Health and Retirement Longitudinal Study from 2011 to 2020
Source: Front Public Health. 2024 Aug 23;12:1416214. doi: 10.3389/fpubh.2024.1416214 (PMC11382495; doi:10.3389/fpubh.2024.1416214)
Supplement: Supplementary file 1 [file Table_1.docx]

Supplementary Material

# Supplementary Figures

Table S1. Effect of healthy aging on fall -unadjusted model.

| **Domains** | **Criteria** | **Variable** | **HR** | **95%Ci** | ***p*-value** |
| --- | --- | --- | --- | --- | --- |
| Absence of Chronic disease | Number of chronic disease | | | | |
|  | ≥1 | Usual aging | 1.02 | (0.97-1.06) | 0.41 |
|  | 0 | HA |  | [ref] |  |
| Good Physical Function | ADL | | | | |
|  | ≥1 | Usual aging | 1.32 | (1.19-1.47) | <0.0001 |
|  | 0 | HA |  | [ref] |  |
|  | IADL | | | | |
|  | ≥2 | Usual aging | 1.32 | (1.25-1.40) | <0.0001 |
|  | 0–1 | HA |  | [ref] |  |
|  | Sum Of ADL And IADL | | | | |
|  | ≥2 | Usual aging | 1.20 | (1.08-1.35) | <0.0001 |
|  | 0-1 | HA |  | [ref] |  |
| Normal Cognitive Function | Cognitive impairment | | | | |
|  | Yes | Usual aging | 1.16 | (1.10-1.23) | <0.0001 |
|  | No | HA |  | [ref] |  |
| Good Psychological Adaptation | Depression | | | | |
|  | Yes | Usual aging | 1.4 | (1.34-1.47) | <0.0001 |
|  | No | HA |  | [ref] |  |
| Active social engagement | Participation in one or more activities | | | | |
|  | Yes | HA |  | [ref] | <0.0001 |
|  | No | Usual aging | 1.23 | (1.18-1.28) |  |

Table S2 Regional comparison of the time-dependent Cox recurrent models between fall and related factors(including a history of falls)

| Variable | Categories | Urban | |  | Rural | |  |
| --- | --- | --- | --- | --- | --- | --- | --- |
|  |  | HR | 95% CI | *p*-Value | HR | 95% CI | *p*-Value |
| Age (years) | 45-54 | 1.00 | [ref] |  | 1.00 | [ref] |  |
|  | 55-64 | 0.76 | (0.63-0.91) | <0.001 | 0.66 | (0.73-0.86) | <0.001 |
|  | ≥65 | 0.74 | (0.61-0.89) | <0.001 | 0.70 | (0.71-0.85) | <0.001 |
| Gender | Male | 0.71 | (0.61-0.83) | <0.001 | 0.75 | (0.67-0.8) | <0.001 |
|  | Female | 1.00 | [ref] |  | 1.00 | [ref] |  |
| Education | Illiteracy | 1.00 | [ref] |  | 1.00 | [ref] |  |
|  | Primary school | 0.91 | (0.80-1.04) | 0.003 | 0.94 | (0.83-0.94) | 0.125 |
|  | Middle school | 0.84 | (0.74-0.97) | <0.001 | 0.80 | (0.68-0.8) | 0.174 |
|  | High school and above | 0.90 | (0.76-1.07) | <0.001 | 0.73 | (0.61-0.78) | <0.001 |
| Marital status | Married | 0.89 | (0.79-1.01) | 0.022 | 0.94 | (0.91-1.04) | 0.13 |
|  | Unmarried/divorced/widowed | 1.00 | [ref] |  | 1.00 | [ref] |  |
| Physical Activity intensity | No | 1.00 | [ref] |  | 1.00 | [ref] |  |
|  | Light | 1.08 | (0.91-1.29) | 0.304 | 1.21 | (1.11-1.34) | <0.001 |
|  | Moderate | 0.97 | (0.82-1.15) | 0.864 | 1.25 | (1.18-1.42) | <0.001 |
|  | Intense | 1.26 | (1.06-1.50) | 0.002 | 1.14 | (1.07-1.28) | <0.001 |
| Smoke | Yes | 1.03 | (0.89-1.19) | 0.999 | 0.98 | (0.93-1.08) | 0.693 |
|  | No | 1.00 | [ref] |  | 1.00 | [ref] |  |
| Drink | Yes | 1.12 | (0.99-1.25) | <0.001 | 1.19 | (1.17-1.32) | <0.001 |
|  | No | 1.00 | [ref] |  | 1.00 | [ref] |  |
| Disability | Yes | 1.52 | (1.31-1.77) | <0.001 | 1.62 | (1.26-1.49) | <0.001 |
|  | No | 1.00 | [ref] |  | 1.00 | [ref] |  |
| Medical insurance | No | 1.00 | [ref] |  | 1.00 | [ref] |  |
|  | Urban employee medical insurance | 0.65 | (0.49-0.84) | 0.001 | 0.72 | (0.63-0.97) | 0.009 |
|  | Urban and rural resident medical insurance | 0.51 | (0.38-0.68) | <0.001 | 0.53 | (0.49-0.68) | <0.001 |
|  | Urban resident medical insurance | 0.61 | (0.46-0.82) | <0.001 | 0.75 | (0.68-1.25) | 0.122 |
|  | New rural cooperative medical insurance | 0.56 | (0.44-0.73) | <0.001 | 0.61 | (0.72-0.94) | <0.001 |
|  | Government medical insurance | 0.73 | (0.47-1.11) | 0.138 | 0.73 | (0.5-1.22) | 0.227 |
|  | Other | 0.56 | (0.34-0.91) | 0.020 | 0.42 | (0.33-0.72) | <0.001 |
| Pension | Yes | 0.77 | (0.69-0.87) | <0.001 | 0.76 | (0.6-0.68) | <0.001 |
|  | No | 1.00 | [ref] |  | 1.00 | [ref] |  |
| Life satisfaction | Yes | 1.08 | (0.98-1.20) | <0.001 | 1.10 | (1.23-1.38) | 0.008 |
|  | No | 1.00 | [ref] |  | 1.00 | [ref] |  |
| Self-reported health | Poor | 1.00 | [ref] |  | 1.00 | [ref] |  |
|  | Normal | 0.71 | (0.64-0.80) | <0.001 | 0.74 | (0.64-0.71) | <0.001 |
|  | Good | 0.51 | (0.44-0.60) | <0.001 | 0.49 | (0.44-0.52) | <0.001 |
| History of fall | Yes | 2.08 | (1.89-2.30) | <0.001 | 2.22 | (0.44-0.52) | <0.001 |
|  | No | 1.00 | [ref] |  | 1.00 | [ref] |  |
| Healthy aging | Yes | 1.00 | [ref] |  | 1.00 | [ref] |  |
|  | No | 1.05 | (0.89-1.23) | 0.452 | 1.15 | (1.00-1.32) | 0.044 |

The results of this table are based on the first two rounds of fall data as the fall history, and sub analysis is conducted starting from the third round of data.

Table S3. Regional Comparison of the Impact of Unhealthy Aging on Falls in Subpopulations of 6,654 Participants With Metabolic Biomarker Measurements

|  | Urban | | Rural | |
| --- | --- | --- | --- | --- |
|  | Unhealthy aging | | | |
|  | HR (95%CI) | *P* value | HR (95%CI) | *P* value |
| Model | 1.05(0.93-1.18) | 0.45 | 1.11(1.01-1.23) | 0.003 |
| Adjusted as model plus |  |  |  |  |
| Total Cholesterol | 0.95(0.75-1.21) | 0.69 | 1.30(1.08-1.57) | 0.006 |
| Triglycerides | 0.96(0.75-1.22) | 0.72 | 1.29(1.07-1.56) | 0.007 |
| HDL cholesterol | 0.95(0.75-1.22) | 0.70 | 1.29(1.07-1.56) | 0.008 |
| LDL cholesterol | 0.95(0.75-1.22) | 0.72 | 1.30(1.08-1.58) | 0.005 |
| Glucose | 0.94(0.74-1.19) | 0.60 | 1.29(1.06-1.55) | 0.009 |
| Blood urea nitrogen | 0.96(0.75-1.22) | 0.72 | 1.30(1.08-1.57) | 0.007 |
| Glycated Hemoglobin | 0.99(0.78-1.26) | 0.93 | 1.31(1.09-1.59) | 0.008 |
| All biomarkers | 0.98(0.78-1.26) | 0.92 | 1.29(1.07-1.56) | 0.004 |


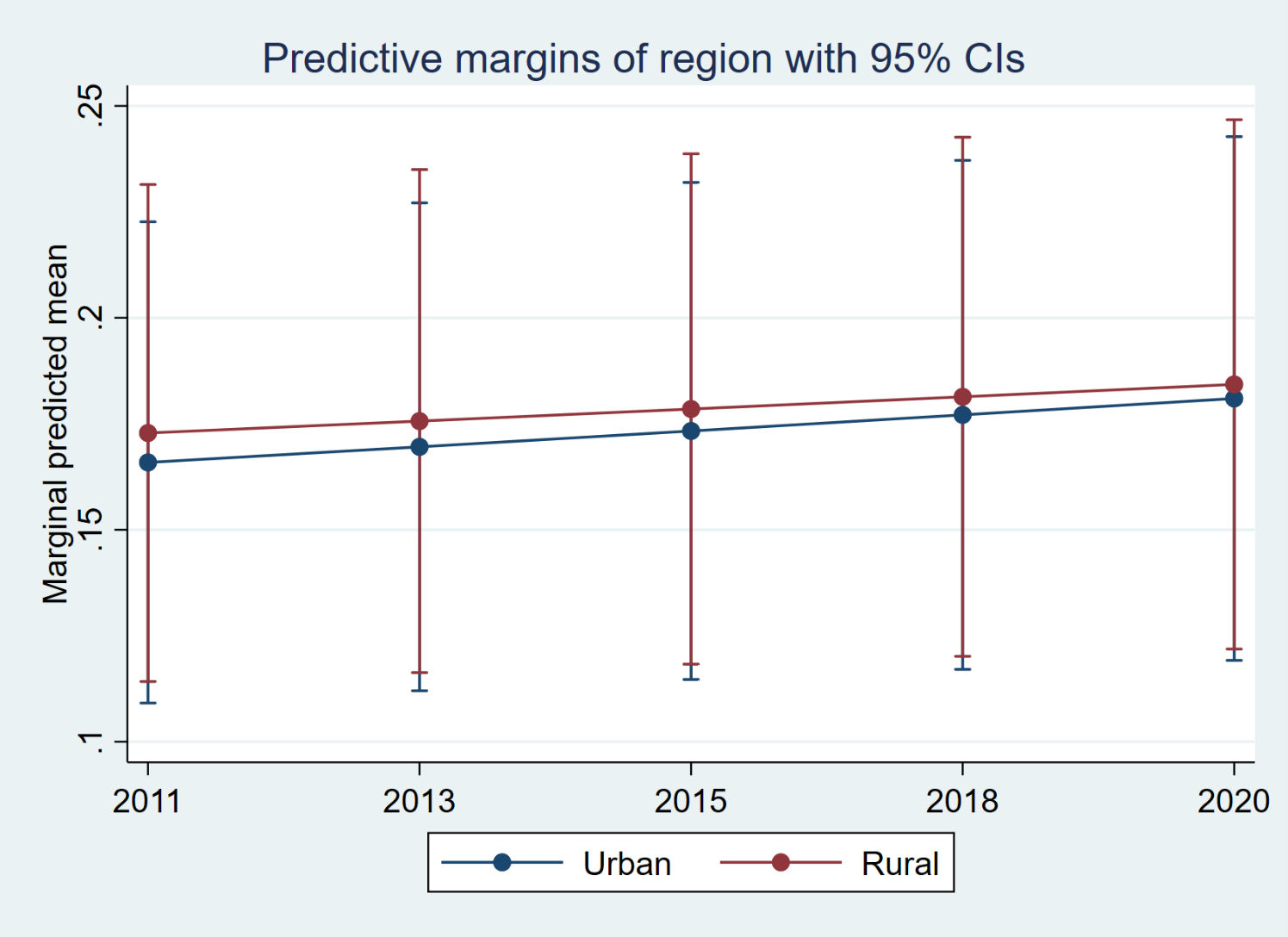


Figure S1：Mixed-effects generalized linear models were used to analyze the fall rates among older adults in urban and rural areas
